# Applications of mixture methods in epidemiological studies investigating the health impact of persistent organic pollutants exposures: a scoping review

**DOI:** 10.1038/s41370-024-00717-3

**Published:** 2024-09-10

**Authors:** Shudi Pan, Zhenjiang Li, Bruna Rubbo, Victoria Quon-Chow, Jiawen Carmen Chen, Brittney O. Baumert, Erika Garcia, Max T. Aung, David V. Conti, Lida Chatzi

**Affiliations:** 1 Department of Population and Public Health Sciences, Keck School of Medicine, University of Southern, California, Los Angeles, CA USA; 2 Department of Dana and David Dornsife College of Letters, Arts and Sciences, University of Southern California, Los Angeles, CA USA

**Keywords:** Chemicals in Products, Pesticides, Perfluorinated Chemicals, Mixtures, Epidemiology, Mixture Analysis Methods

## Abstract

**Background:**

Persistent organic pollutants (POPs) are environmental chemicals characterized by long half-lives in nature and human bodies, posing significant health risks. The concept of the exposome, encompassing all lifetime environmental exposures, underscores the importance of studying POP as mixtures rather than in isolation. The increasing body of evidence on the health impacts of POP mixtures necessitates the proper application of statistical methods.

**Objectives:**

We aimed to summarize studies on the overall effects of POP mixtures, identify patterns in applications of mixture methods—statistical methods for investigating the association of mixtures—and highlight current challenges in synthesizing epidemiologic evidence of POP mixtures on health effects as illustrated through a case study.

**Methods:**

We conducted a systematic literature search on PubMed and Embase for epidemiological studies published between January 2011 and April 2023.

**Results:**

We included 240 studies that met our eligibility criteria. 126 studies focused on per- and polyfluoroalkyl substances (PFAS) mixtures only, while 40 analyzed three or more classes of POPs in mixture analyses. We identified 23 unique mixture methods used to estimate the overall effects of POP mixtures, with Bayesian Kernel Machine Regression (BKMR), a type of response-surface modeling, being the most common. Additionally, 22.9% of studies used a combination of methods, including response-surface modeling, index modeling, dimension reduction, and latent variable models. The most extensively explored health outcome category was body weight and birth sizes (*n* = 43), and neurological outcomes (*n* = 41). In the case study of PFAS mixtures and birth weight, 12 studies showed negative associations, while 4 showed null results, and 2 showed positive associations.

**Impact Statement:**

- This scoping review consolidates the existing literature on the overall effects of POP mixtures using statistical methods. By providing a comprehensive overview, our study illuminates the present landscape of knowledge in this field and underscores the methodological hurdles prevalent in epidemiological studies focused on POP mixtures. Through this analysis, we aim to steer future research directions, fostering a more nuanced comprehension of the intricate dynamics involved in assessing the health effects of POP mixtures. Our work stands as a significant contribution to the ongoing exploration of the chemical exposome.

## Introduction

Persistent organic pollutants (POPs) are environmentally and biologically persistent synthetic chemicals widely used in manufacturing such as flame retardants and pesticides [1–3]. Previous research on POPs spans various classes of chemicals, and the following classes have been commonly investigated due to their widespread presence and potential adverse effects on human health and environment, including organochlorine pesticides, polychlorinated biphenyls (PCBs), polybrominated diphenyl ethers (PBDEs), polychlorinated dioxins and furans (PCDD/Fs) and per- and polyfluoroalkyl substances (PFASs) [4]. Most POPs were prohibited from usage after the Stockholm Convention in 2001 [5]. However, traceable levels of POPs have still been observed across the globe and even in regions where people have never used POPs [2]. The general population is exposed to POPs through multiple routes, such as inhalation, contaminated drinking water and diet [6–8]. Previous studies have found that POPs act as both endocrine disruptors and oxidative stress inducer [9], associated with various adverse health outcomes, such as cardiovascular diseases, metabolic diseases [10, 11], liver injury [12], neurodevelopment [13], reproductive outcomes [14, and cancer [15].

The existing evidence on the health risks of exposure to POPs has been gradually moving away from single pollutant analysis to a mixture-based approach. Mixture is defined as a combination of at least three individual chemicals that are designated for a specific purpose [16, 17]. This shift is in alignment with the conceptual framework for exposome research. The exposome framework aims to quantify “the totality of human environmental exposures from conception onwards,” which underscores the need to shift our perspective from traditional “one-exposure-one-disease” to a focus on multiple environmental exposures and their overall effects [18, 19]. In fact, humans are exposed to multiple chemicals simultaneously at any given time point and cumulatively across their lifetimes. Moreover, chemicals are highly correlated and many come from the same source, potentially having antagonistic or synergistic effects in mixtures [20]. This is particularly critical for POPs. Unlike other environmental pollutants, POPs are known for long half-lives in human body, leading to potential interactions that can be antagonistic or synergistic when mixed with each other or other chemicals. Classic single-pollutant models fail to capture the complexity of correlated exposures and cannot accurately delineate the interaction and overall effects of POP exposures. Methods that investigate the POPs mixture effects are imperative for a comprehensive overview of the health effects of POPs.

In the past two decades, new statistical methods have been developed to better understand the complex relationships between environmental chemical mixtures and health outcomes and to better address some of the statistical challenges, such as multicollinearity [21]. Methods, which include Bayesian kernel machine regression (BKMR) [22], weighted-based g-computation [23], and weighted quantile sum regression (WQS) [24], were developed to model chemical mixtures. These methods are able to address these following research questions: 1) the overall effect of chemical mixtures; 2) the effect of interactions among mixture components; 3) the relative toxic effect of individual agents in the mixtures; and 4) the specific patterns of exposures in the population [25, 26]. However, few methods can answer these research questions simultaneously, and many previous studies on POP mixtures focus on the overall mixture effects as a primary research question. Overall effects are defined as the effects of chemicals mixtures combined to characterize the total health burden of environmental mixtures. When individual compounds are below regulatory concentrations, there may still be significant combined overall effects on shared health endpoints [26]. Moreover, given that new chemicals are continuously being created and many POPs exhibit similar health effects via a common mechanism [2], a better understanding of the overall effects of POP mixtures is needed for risk stratification and future intervention based on chemical mixtures.

To our knowledge, no previous research has comprehensively reviewed the overall effects of exposures to POPs on health outcomes. The objective of this review is to identify current methodological approaches used to evaluate the overall effects of POP mixtures and to highlight the current challenges and obstacles encountered in synthesizing the overall effects of POP mixtures, A case study focusing on the most extensively studied POP mixtures and their health outcome was also included to detail the challenges.

## Methods

This scoping review was written following the Preferred Reporting Items for Systematic Reviews and Meta-Analyses Extension for Scoping Reviews (PRISMA-ScR) [27]. We registered our protocol at Open Science Framework (OSF) with registration number k8xe6 [28].

### Search strategy

The search strategy was developed based on the Population Exposure Outcomes and Results (PEOR) framework (Table 1). We conducted systematic literature searches in electronic databases Embase and MEDLINE of PubMed in early August 2022 and repeated in April 2023. The search strategies were reviewed by an experienced librarian and the entire research team. Specifically, we focused on five classes of POPs, which were 1) organochlorine pesticides (OCPs) such as Dichlorodiphenyltrichloroethane (DDT) and its metabolites, 2) polybrominated diphenyl ethers (PBDEs), 3) polychlorinated biphenyls (PCBs), 4) per- and polyfluoroalkyl substances (PFASs), and 5) polychlorinated dibenzo-p-dioxins (PCDDs) and polychlorinated dibenzofurans (PCDFs), abbreviated as PCDD/Fs. We chose to focus on these five chemical classes of POPs due to their widespread exposures worldwide and persistence in nature and biological systems. They were also listed as targeted chemicals in the Stockholm convention [29]. Detailed search terms were included in Supplementary Table S1. We searched journal studies published between January 1^st^ 2011 and April 26^th^, 2023, and no language restriction was imposed. The publication year was restricted to 2011–2023 because the majority of mixture methods were developed in recent years.

**Table 1:** Population, exposure, outcome, results (PEOR) framework.

PEOR elements	Type of evidence
Population (P)	Human; any population
Exposure (E)	Measured occurrence of the environmental exposure to a mixture of at least three individual POPs with or without other chemical or non-chemical stressors via biomonitoring**;** Five classes of POPs were focused and those were: Organochlorine pesticides, polybrominated diphenyl ethers, polychlorinated biphenyls, per- and polyfluoroalkyl substances and polychlorinated dibenzo-p-dioxins/dibenzofurans
Outcome (O)	Any type of effects on human health
Results (R)	Overall effects of POP mixtures evaluated by statistical methods identified a priori

*POPs* persistent organic pollutants.

We built a search strategy with two main facets: “POP mixture exposures” and “mixture methods”. For the exposures to POP mixtures, we included both Medical Subject Headings (MeSH) in PubMed or exploded terms in Embase, and individual common chemicals names as keywords in the search strategy. Individual chemicals were searched both as MeSH terms and keywords to capture as many journal studies related to exposures to POPs as possible. Chemical names were searched in both full names and acronyms if applicable (i.e. hexachlorobenzene and HCB). Chemicals such as Dichlorodiphenyl Dichloroethylene (DDE) have multiple synonyms as full names. In such case, we decided to keep full names used in ATSDR toxicological profiles instead of including all synonyms for simplicity [30]. We did not include any full names of individual PCBs and PBDEs because PCBs and PBDEs are conventionally used as acronyms in literature. All chemical names were cross-referenced with PubChem to ensure accuracy [31]. The second part of the search strategy is overall effects of POP mixtures using mixture methods. To develop a search strategy that would capture all related mixture analysis studies with high sensitivity, we first conducted a pilot search to identify statistical methods commonly employed for estimating the overall effects of persistent organic pollutants (POPs). The search terms used were "mixture*" OR "chemical mixture*" OR "overall effect*" OR “overall association*" OR “overall exposure*" OR “cumulative effect*" OR “combined effect*" OR “joint impact*" OR “joint exposure*” OR “joint effect*” OR “multi-pollutant” OR multipollutant. We compiled the mixture methods identified through the pilot search along with mixture methods identified through previous reviews as keywords used in search terms [21, 26, 32]. With this multifaceted search strategy, the majority of the studies suitable for this review were identified to the best of our knowledge.

We complemented the search by conducting a hand search in relevant journals and scanned the reference lists of related reviews.

### Eligibility criteria

The inclusion and exclusion criteria were formulated based on the PEOR framework (Table 1). We included peer-reviewed studies which were: 1) conducted in human subjects; 2) used any type of observational study design such as cohort, case–control and cross-sectional studies; 3) evaluated at least three POPs based on the National Institute of Environmental Health Science (NIEHS) definition of mixture in biomonitoring [33]; 4) estimated overall effects of POP mixtures using statistical methods. Statistical methods included were mixture methods identified in the previous literature [20, 21] or methods we identified through the two-stage literature screening approach. The final list of methods used for literature search were: WQS, BKMR, quantile g-computation, PCA, factor analysis, clustering methods such as k-means and hierarchical clustering, exposure continuum mapping, latent class/profile analysis, and other Bayesian approaches related to environmental mixture overall effects. We also included novel mixture methods developed through the Powering Research Through Innovative Methods for Mixtures in Epidemiology(PRIME) workshop [21] (Methods were listed in Supplementary Table S1). We excluded journal studies based on the following criteria: 1) studies conducted in animals or in silico, in vitro studies; 2) POP mixtures in studies were measured in air, food, drinking water, occupational settings or direct administration; 3) studies only used summation measures of POPs; 4) studies only used non-statistical toxicology-based studies such as toxic equivalency factors for PCDD/Fs [34]; 5) sources considered as gray literature, reviews, pre-prints, conference proceedings manuscripts, editorials, websites, or textbook chapters.

### Study selection

We conducted our literature screening in Covidence (Covidence systematic review software, Veritas Health Innovation, Melbourne, Australia). Two reviewers (SP and VQ) independently screened titles and abstracts, and any discrepancies were discussed with a third reviewer (BR). For all journal studies that met the eligibility criteria from the title and abstract screening, two reviewers (SP and BR) independently screened the full texts to determine the eligibility based on the pre-specified criteria (Supplementary Table S2).

### Data extraction

Three independent reviewers (CC, SP and VQ) extracted information using a standardized data extraction form. Two reviewers were randomly assigned to each paper, and discrepancies were resolved in discussions among all three reviewers. We extracted and recorded information from each study using a pre-specified data extraction form, including titles, authors, publication year, study country, study site, health outcome categories, study design, study population, sample size, age, exposure matrix, POP exposures included in each mixture methods, mixture methods, outcomes, and covariates (Supplementary Table S3). Health outcome categories were developed based on PFAS systematic evidence map studies, and individual health outcomes under each categories were provided in Supplementary Table S4 [35, 36]. The categories were: Body weight, size & growth, cancer, cardiometabolic, cardiovascular, dermal, endocrine, hepatic, immune, metabolic, mortality, musculoskeletal, nervous, reproductive, respiratory, urinary and systemic biomarkers. Biomarkers were categorized based on their association with specific health outcomes; for example, liver biomarkers were classified under “Hepatic.” Conversely, biomarkers indicative of nonspecific processes, which are linked to multiple health outcomes, were designated as “systemic biomarkers.” We did not provide a summary of main findings for each study due to the heterogeneity of the health outcomes. As a proof-of-concept for the interpretations of mixture methods results, we extracted the information of results in studies estimating the overall effects of the most widely investigated exposure and the most widely investigated health outcome.

### Data synthesis

We first harmonized chemical names to keep chemical names consistent across studies (Supplementary Table S5). We grouped the class of POPs for all included studies and summarized the frequency of each statistical method used. Methods that appeared twice or more were also grouped based on their modeling strategy We provided a narrative synthesis for the overall impact of the associations of the most widely investigated exposure and the most widely investigated health outcome to identify knowledge gaps in the interpretations of epidemiologic findings of POP mixtures.

## Results

### Selected sources of evidence

**Fig. 1:**
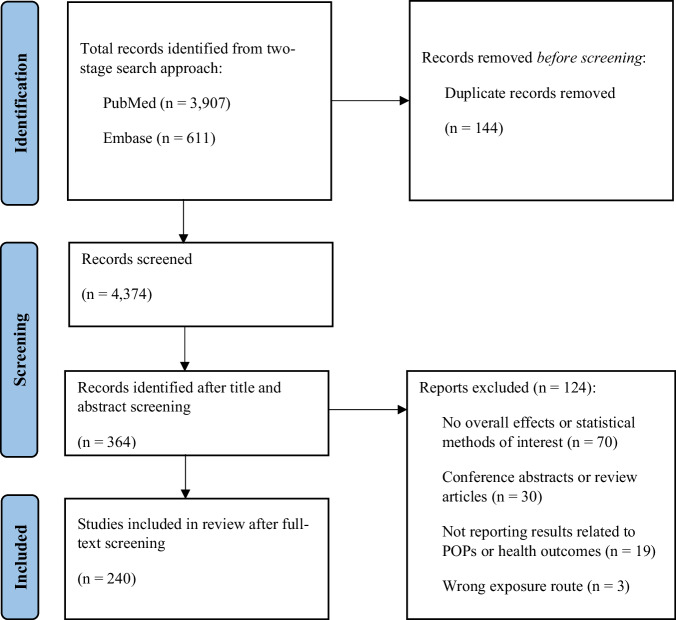
PRISMA diagram of literature screening. The workflow of literature screening in the current study. We adopted the PRISMA diagram for the systematic search in PubMed and Embase, detailing the number of abstracts screened and the full texts retrieved.

A total of 3907 records were identified from PubMed and 611 records were identified from Embase from searches published between January 2011 to April 2023 (Fig. 1). After removing 144 duplicate records, we reviewed title and abstracts of 4374 total records for relevance. We reviewed the full-text of 364 studies using pre-set inclusion and exclusion criteria (i.e. excluding animal studies, see Table 2). A further 124 studies were removed during the full-text screening due to various reasons (e.g. no overall effects or statistical methods of interest). We included 240 unique studies in this review.

**Table 2:** Summary of the study characteristics by POP classes included in the review.

Characteristic	Overall, *N* = 240^a^	PFAS, *N* = 126	PBDE, *N* = 14	OCP, *N* = 12	PCB, *N* = 9	PCB + PCDD/Fs, *N* = 7	OCP + PCB, N = 22	OCP + PBDE, *N* = 1	PFAS with another class, *N* = 9	Multiple class^b^, *N* = 40
**Study design**										
Cohort study	130	65	7	7	5	1	10	0	8	27
Cross sectional study	83	48	5	2	2	6	9	1	1	9
Case–control study	27	13	2	3	2	0	3	0	0	4
**Country/Regions in which the study was conducted**										
United States	97	48	10	1	6	6	8	0	3	15
China	74	59	3	7	1	0	1	1	1	1
Europe	46	15	0	0	1	0	6	0	4	20
Canada	9	1	1	0	0	0	5	0	1	1
South Korea	4	1	0	0	0	0	1	0	0	2
Japan	3	0	0	1	1	1	0	0	0	0
Australia	2	1	0	0	0	0	0	0	0	1
Columbia	1	0	0	1	0	0	0	0	0	0
Mexico	1	0	0	0	0	0	1	0	0	0
Saudi Arabia	1	0	0	1	0	0	0	0	0	0
Singapore	1	1	0	0	0	0	0	0	0	0
South Africa	1	0	0	1	0	0	0	0	0	0
**Population characteristics**										
Mother-child pairs	78	37	5	8	2	1	3	0	6	16
General population	74	41	6	0	3	6	10	0	1	7
Pregnant women	32	19	1	1	1	0	2	0	1	7
Patients	20	7	1	0	2	0	3	0	0	7
Children	18	10	1	1	0	0	1	1	1	3
Adolescents	9	5	0	1	0	0	3	0	0	0
Residents living near contaminated areas	6	5	0	1	0	0	0	0	0	0
Occupational population	3	2	0	0	1	0	0	0	0	0
**Sample size**										
<= 100	12	2	1	0	1	0	1	1	0	6
100–500	105	37	8	9	3	2	15	0	6	25
500–1000	50	36	1	2	2	1	4	0	0	4
> 1000	73	51	4	1	3	4	2	0	3	5
**Publication year**										
Before 2016	10	0	0	1	2	1	1	0	1	4
2016–2020	43	12	3	5	3	2	3	0	2	13
2021	54	24	4	3	1	0	8	0	5	9
2022	77	49	4	2	3	3	5	0	1	10
2023	56	41	3	1	0	1	5	1	0	4

Chemical class: individual chemicals in each POP class from the 240 included studies are listed in Supplementary Table S6.

*PFAS* Per- and polyfluoroalkyl substances, *PBDE* Polybrominated diphenyl ethers, *OCP* Organochlorine pesticides, *PCB* Polychlorinated biphenyls, *PCDD/Fs* polychlorinated dibenzo-p-dioxins (PCDDs) and polychlorinated dibenzofurans (PCDFs).

^a^Mixture components other than POPs were not included in this review.

^b^Three or more than three POP class.

### Characteristics of included studies

Detailed information regarding study characteristics of included studies is in Supplementary Table S6. We grouped the 240 studies based on their POP chemical classes and summarized their study characteristics in Table 2. The majority of the studies examined PFAS as the only POP mixtures of interest (*n* = 126), while fourteen studies focused on PBDEs only, 12 studies on organochlorine pesticides, and nine studies on PCB mixtures. For the POP class combinations, seven studies focused on the PCBs with PCDD/Fs, of which all included both dioxin-like and non-dioxin like PCBs (Supplementary Table S6). There were twenty-two studies that examined a mixture of organochlorine pesticides with PCBs and one study of organochlorine pesticides with PBDEs. In addition, 9 studies evaluated PFAS mixtures along with another POP class (PCBs, PBDEs, OCPs or PCDD/Fs). 40 studies looked at three or more POP classes, and some studies also included heavy metals or other non-persistent chemicals as components in mixture analysis (*n* = 40).

The majority of studies were cohort studies (*n* = 130). However, 83 relied on cross-sectional data, with fewer studies using a case–control design (*n* = 27). Most studies were conducted in the United States (*n* = 97) or in China (*n* = 74). Multiple studies were conducted in Europe (*n* = 46), Canada (*n* = 9), South Korea (*n* = 4), Japan (*n* = 3), and Australia (*n* = 2). A single study was identified in each of the following countries: Colombia, Mexico, Saudi Arabia, Singapore, South Africa. For studies conducted in the US, about half of the studies (*n* = 48/97, 49%) focused on PFAS mixtures only. Seventeen studies evaluated the other POP chemical classes only and 15 studies evaluated multiple classes of POPs. The majority (*n* = 59/74) of studies in China evaluated PFAS mixtures only and one study evaluated PFAS, PCB with PBDEs. 20 of European studies evaluated three or more POP classes and 15 European studies focused on PFAS mixtures alone.

Seventy-eight studies had a birth cohort design and 32 focused on pregnancy women. There were also 18 studies focusing on children and 9 studies focusing on adolescents. Seventy-four studies examined POP exposures in general populations with a wide age range from mid-aged to elderly populations (Supplementary Table S6). In addition, some studies focus on patients from hospital-based study designs (*n* = 20), residents living near contaminated areas (*n* = 6), or occupational populations such as military, employees from government organization and retired workers (*n* = 3).

About half of the studies (*n* = 105/240) included 100–500 people. There were 73 studies with more than 1000 people included in their analysis. There has been a notable noticeable rise in the number of published studies in recent years (Table 2).

### Overview of mixture methods applied in estimation of overall effects of POP mixtures

**Fig. 2:**
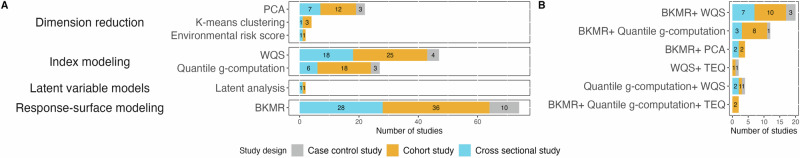
Summary of mixture methods used in overall effect estimation from included studies by study design. Panel **A** is the frequency of mixture methods in studies that applied one mixture method grouped by modeling strategy and study design. Mixture methods appeared only once in studies were excluded. Panel **B** is the frequency of mixture methods combination in studies that applied two or more mixture methods categorized by study design. Mixture methods combination appeared only once in studies were excluded. PCA Principal component analysis, WQS Weighted quantile sum, BKMR Bayesian kernel machine regression, TEQ toxicity equivalent quantity.

We identified a total of 23 unique mixture methods used to estimate the overall effects of POP mixtures in relation to health outcomes. For better comparison. Bayesian weighted sum, Bayesian WQS, Weighted quartile score methods were grouped as WQS. Latent profile analysis and latent factor analysis were grouped as latent analysis. 185 studies used a single mixture method (Fig. 2A), whereas 55 studies (Fig. 2B) used at least 2 mixture methods. For the 185 studies that used single mixture methods, the top 4 mixture methods used were BKMR, WQS, quantile g-computation and PCA (*n* = 74, 47, 27 and 22 respectively). K-means clustering was used in four studies. Environmental risk score and latent analysis were also applied in two studies. BKMR, WQS, quantile g-computation, environmental risk scores are supervised, and the rest of the methods are unsupervised. We also summarized methods that were only used once in the included studies. These are Bayesian factor analysis, Bayesian hierarchical modeling approach with g-computation (BHRM-g) [37], Bayesian joint latent class [38], Bayesian structure additive regression models with spike-slab priors [39], Exposure continuum mapping (ECM) [40], factor analysis [41], first principal direction of mediation (PDM) [42], latent class model [43], latent profile model [44], Sparse PCA [45], super learner and g-computation, and weighted kernel machine regression methods [46]. For the 55 studies that applied two or more mixture methods, researchers used different combinations of modeling strategies. Most mixture methods can be classified as response-surface modeling, index modeling, dimension reduction and latent variable models. Index modeling produced a readily interpretable index, which is a weighted average of exposure mixtures and used to regress against health outcomes of interest [21, 47]. Latent variable models share similar characteristics but are more often used for predefined exposure groups [21, 48]. Dimension reduction methods were used when many exposures are present in studies, to reduce multidimensional exposures to orthogonal components or other summarized scores to represent the POP mixtures that can be regressed against health outcomes of interest. Response-surface modeling fit a multidimensional space of the exposure data to model non-linear effects of chemical exposures [32]. The most popular pair is response-surface modeling with index modeling, to combine the advantages of both methods. BKMR was most frequently used with WQS (*n* = 20), followed by BKMR with quantile g-computation (*n* = 12).

**Fig. 3:**
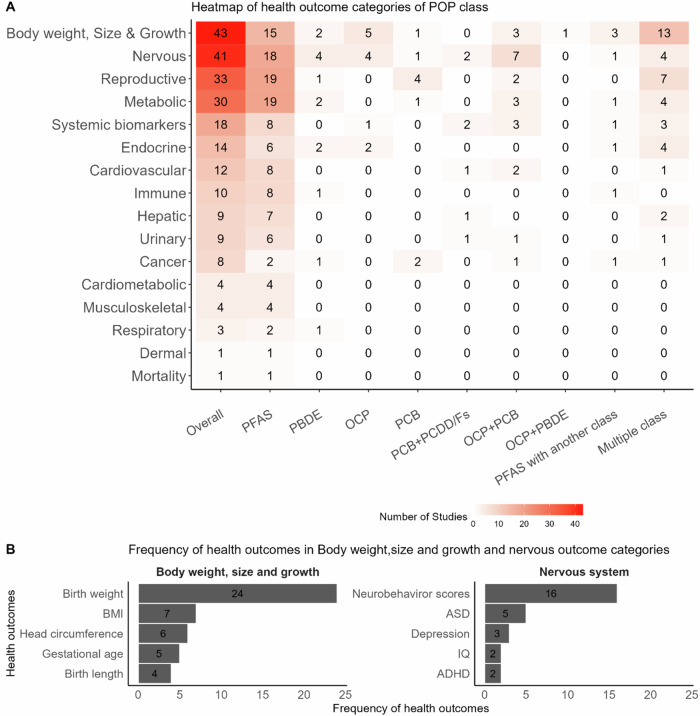
Summary of health outcome categories of the included studies. **A** Heat map of health outcome categories by POP class in the included studies. Numbers represent the number of publications where the associations between POP class (rows) and health outcome categories (columns) were examined. Note: Two studies examined two outcome categories together (endocrine and metabolic, hepatic and metabolic). **B** The five most frequently observed health outcomes of the “Body weight, size and growth” and “Nervous” outcome categories. Note: Each study included multiple outcomes. Chemical class: individual chemicals in each POP class from the 240 included studies are in Supplementary Table S6. POP persistent organic pollutants, PFAS per- and polyfluoroalkyl substances, PBDE polybrominated diphenyl ethers, OCP organochlorine pesticides, PCB polychlorinated biphenyls, PCDD/Fs polychlorinated dibenzo-p-dioxins (PCDDs) and polychlorinated dibenzofurans (PCDFs), BMI body mass index, ADHD attention-deficit/hyperactivity disorder, ASD autism spectrum disorder, IQ intelligence quotient.

The mixture methods were also categorized based on study design (Fig. 3), sample size and publication years (Supplementary Fig. S1) and we found that the choices of mixture methods were not influenced by the sample size, publication year, and study design of included studies.

Researchers used different strategies to reduce the dimensionality of POP mixtures as a pre-modeling step before applying mixture methods. For example, in addition to including compounds individually in mixture modeling, researchers usually combined PFOS, PFOA isomers as summation measures as components in models [49], and some studies also included summation of POP as constituents in mixture modeling [50, 51]. For PCBs, two studies also used the summation measure of 4 PCBs (PCB118, PCB138/158, PCB153, PCB180) from the New Bedford cohort and HOME cohort [52–57], while some studies used the summation measure of 3 PCBs (PCB138, PCB153, PCB180) for the same cohort in Faroe Islands [46, 58, 59], and some studies used summation measures of ten PCBs [60, 61]. We also identified studies utilizing hierarchical clustering methods to group chemicals and used summed measures of chemical clusters in multiple linear regression [62]. In addition, although we excluded studies which only used Toxic Equivalency Factors (TEFs) or other toxicology-based methods, some of the included studies applied TEFs to summarize TCDD and then used as components in mixture methods [63].

### Overview of health outcomes of included studies

The individual health outcomes examined in each study were grouped by health outcome categories and listed in Supplementary Table S4. The most extensively explored health outcome category was body weight and birth size (*n* = 43 Fig. 3) and neurological outcomes (*n* = 41). In relation to the POP class (Fig. 3A), the health-related outcomes most extensively explored in studies with PFAS as mixtures were metabolic (*n* = 19), reproductive (*n* = 19), and nervous health outcome categories (*n* = 18). For studies analyzing multiple POP classes, growth outcomes such as birth weight were most frequently used (*n* = 13), followed by reproductive outcomes (*n* = 7).

Most of the studies on developmental outcomes consisted of mother-child pairs or adolescent groups. Within birth cohort studies, developmental outcomes of the child were measured such as birth weight (*n* = 24), head circumference (*n* = 6), birth length (*n* = 4), and gestational age (*n* = 5). Among children and adolescents, BMI was measured and used as an outcome in seven studies (Fig. 3B).

For neurodevelopment outcomes, neurobehavior scores were used 16 times in studies, following with autism spectrum disorder (ASD) (*n* = 5), depression (*n* = 3), IQ (*n* = 2), and ADHD (*n* = 2) (Fig. 3C).

### Case study: synthesis of the overall effects of PFAS mixtures and birth weight

Due to the heterogeneity of the health outcomes, we provided narrative synthesis of the overall effects of PFAS mixtures on birth weight and investigated how different statistical methods yield various results. For studies that evaluated POP classes in addition to PFAS, we only synthesized the results regarding PFAS mixtures.

Among the 18 studies on the association between PFAS mixtures and birth weight, 12 used one mixture methods, while 6 studies applied two or more mixture methods (Table 3). Various modeling strategies have been applied to examine the overall effects of PFAS mixture and birth weight, with index modeling as the most prevalent strategy, using methods such as WQS and quantile g-computation. Studies with two or more mixture methods generally used a combination of modeling strategies, and the most applied combination was response-surface modeling with index modeling. In addition, 8 studies estimated PFAS mixtures only, while the rest of the studies included either chemicals from other POP classes or other chemical or non-chemical stressors as components in mixture modeling (Table 3). Twelve studies found negative associations while 4 studies found null associations between PFAS mixtures and birth weight. For non-linearity of the overall effects of PFAS mixtures, most studies reported monotonic relationships while one study reported an U-shaped relationship between PFAS mixtures and birth weight [39]. No specific chemical-chemical interactions were reported for the effects of PFAS mixtures on birth weight.

**Table 3:** Narrative synthesis of the association between PFAS mixtures and birth weight with different mixture methods.

Reference	Modeling strategy	Mixture methods	Other components^a^	Association direction
**Single methods**				
Govarts 2016 [58]	Dimension reduction	PCA	OCP, PCB, heavy metals, phthalates, particulate matter	Negative
Gao 2022 [83]	Index modeling	WQS	No	Negative
Padula 2023 [84]		Bayesian weighted sum	No	Negative
Svensson 2021 [61]		WQS	OCP, phthalates, plasticizer, antibacterial, phenols, PAH, pesticides	Negative
Wang 2023 [85]		WQS	No	Null
Shen 2022 [86]		Quantile g-computation	No	Null
Song 2023 [87]		Quantile g-computation	No	Negative
Hu 2021 [88]	Response-surface modeling	BKMR	OCP, PCB, heavy metals, phthalates, phenols	Negative
Wang 2023 [89]		BKMR	No	Positive
Lazarevic 2022 [39]		Bayesian structured GAM w/ spike–slab	No	Null
Luo 2021 [90]		BKMR	No	Negative
Pearce 2021 [40]		Exposure continuum mapping (ECM)	PBDE, OCP, PCB	Positive
**Multiple methods**				
Eick 2022 [69]	Response-surface modeling + index modeling	BKMR + Quantile g-computation	perceived stress and depression	Negative
Eick 2023 [68]		BKMR + Quantile g-computation	perceived stress, depression, gendered racial stress and anxiety	Negative
Marks 2021 [91]		BKMR + WQS	OCP, PCB	Negative
Kalloo 2020 [92]	Dimension reduction	PCA + K-means clustering	PBDE, OCP, PCB, phenols, parabens, phthalates, diammonium phosphate, pyrethroid insecticides, cotinine	Null with one PC negative
Zhuang 2021 [93]	Response-surface modeling + dimension reduction	BKMR + Bayesian factor analysis	PCB	Negative
Eick 2023 [94]	Response-surface modeling + index modeling + dimension reduction	BKMR + Quantile g-computation + PCA	PBDE, OCP, bisphenols, pyrethroid insecticides, phthalates and marijuana and tobacco	Negative

*PFAS* Per- and polyfluoroalkyl substances, *PBDE* Polybrominated diphenyl ethers, *OCP* Organochlorine pesticides, *PCB* Polychlorinated biphenyls, *WQS* Weighted quantile sum, *BKMR* Bayesian kernel machine regression, *PCA* Principal component analysis.

^a^Other components included in mixture analysis models.

## Discussion

### Main findings

To our knowledge, this is the first scoping review on the overall effects of POP mixtures with an emphasis on the application of mixture methods. We included a total of 240 studies, which applied 23 unique mixture methods to estimate the overall effects of POP mixtures on health outcomes. With an increasing number of studies that estimate overall effect of POP mixtures, substantial heterogeneity in the pre-selection of mixture components and the use of mixture methods exists across the 240 reviewed studies. The majority adopted a single chemical-class approach, while 40 studies considered at least 3 classes of POPs simultaneously in their mixture methods analysis. Response-surface modeling such as BKMR was the most frequently used method to estimate the overall effects of POP mixtures. However, we found that the choices of mixture methods were not influenced by the sample size, publication year, and study design of included studies.

In this review, overall effects were defined as cumulative environmental burdens of POP mixtures. As discussed previously, the selection of chemicals included as components was based on various factors and thus estimated overall effects were highly context-based and might not reflect the total burdens of POP mixtures on health outcomes.

Currently, the most abundant studies were on the PFAS mixtures in relation to reproductive, metabolic, nervous, and developmental health outcome category with the most frequently analyzed outcome being birth weight. For the association between PFAS mixture and birth weight, the associations were mostly inverse. However, synthesis regarding overall effects of POP mixtures risks on birth weight showed that it remains challenging to quantitatively synthesize results of single chemical-class POP mixtures across studies. The pre-selected PFASs in PFAS mixtures and employed modeling strategies vary considerably across the eighteen included studies, which underscores the need for standardized meta-analysis protocols to estimate the overall effects of single-class POP mixtures. Here, we developed a list of challenges when synthesizing overall effects and applications of mixture methods in POP mixture as well as provided recommendation for future mixture studies in POPs.

### Challenges in overall effects of POP mixtures

We identified several challenges in estimating the overall effects of POP mixtures: 1) choice of mixture components included in mixture analysis modeling; 2) choice of mixture methods used for modeling; 3) reporting standards and interpretation of mixture methods results pertaining to the epidemiologic research question and risk assessment and 4) incorporation of chemical-chemical interaction.

#### Choice of chemicals in mixture components of modeling

To investigate the joint action of chemical mixtures, researchers have adopted various strategies to select chemicals as components in mixture methods for estimating overall impact of POP mixtures. This selection is motivated by constraints in sample size, availability of biological samples or the logistic aspects of laboratory analyses, which hinders statistical power and model complexity and makes it currently unrealistic to include all POPs as components in mixture analyses.

The selection and incorporation of individual chemicals as components in estimating overall effects from biomonitoring studies are initially constrained by exposure assessment. Typically, chemical exposure assessment focuses on pollutants with recognized concerns or those measurable via targeted methods [25]. Furthermore, studies often apply specific thresholds of detection rate to determine the inclusion of chemicals in mixture modeling. Detection rate is defined as the percentage of samples with a concentration higher than the limit of detection vs. the total analytical samples. Different thresholds of detection rates have been applied, with a broad range from 30% to 85% [64, 65]. These chemical choices stemming from the limit of detection in exposure assessment based on biomonitoring data can result in observational bias such as the streetlight effect [25, 32]. The streetlight effect describes the observational bias toward focusing on certain chemicals due to the availability of data and existing measurement methods, overlooking less familiar but equally hazardous chemicals. It also introduces complexities in comparing overall effects of POP mixtures across studies due to the inconsistency of chemicals included in POP mixtures. Another challenge in estimating overall effects of POP mixtures from biomonitoring studies is the inconsistency of biological matrix and timing of exposure assessment. Most studies on POP mixtures used single serum or plasma samples as the preferred biological matrix. Given POP have long half lives in the human body, blood samples assessed at a single time point serve as a valid proxy to long-term internal dose of POP mixture exposures. However, some studies also included other chemical or non-chemical stressors in overall effect estimation of POP mixtures. These stressors have shorter half-lives and higher intraindividual variability, which often require repeated measurement to capture the long-term exposure windows like the exposure windows of POP mixtures [32]. Previous reviews have suggested the various degrees of exposure misclassification from different classes of chemicals might lead to differential bias in multi-pollutants models [32]. Therefore, caution should be exercised when selecting various stressors from biospecimens as components in the overall effects estimation of environmental pollutant mixtures. A potential solution for addressing measurement error arising from inconsistency of biological matrix is latent variable models [48]. Latent variable models create latent variables between biological matrix to adjust for measurement errors. Previous studies on mercury exposures used structural equation modeling by allowing correlations between maternal hair and cord measured exposures [66].

More than half of the studies selected chemicals in mixture methods modeling based on chemical classes. This is in alignment with the current risk assessment suggestions to regulate chemicals based as chemical classes [67]. The emphasis on chemical class-based categorization in studies is rooted in the pursuit of understanding common biological mechanisms of action. Chemicals within the same class typically share analogous structural features and functional properties, resulting in similar interactions within biological systems [67]. As a complement to mixture methods modeling, strategies have been used for combining chemicals from the same class in overall effect estimations of POP mixtures. For example, aggregated variables of chemicals such as raw or weighted sums based on toxicological properties of chemicals (i.e. Toxicity equivalent quantity (TEQ)) were used to generate mixture components for POPs from the same class in mixture methods. Studies that assessed multiple classes of POPs share similar reasoning as studies based on single chemical class, aiming to elucidate common biological mechanisms of action of POP mixtures. For example, POPs which were endocrine disrupting chemicals (EDCs) are usually grouped together in mixture analysis to provide a comprehensive understanding of health impacts since these POPs share common biological mechanisms. POPs, that were EDCs, were often grouped with other EDCs such as phthalates, phenols, parabens as indicated in our case study on the overall effects on birth weight.

The exposome approach, advocating a shift from singular chemical or chemical class-focused investigations, urges researchers to assess the effects of jointly acting mixtures that encompass both chemical and non-chemical stressors. We found several studies incorporated the exposome perspective to include non-chemical components such as stress and anxiety in the association between POP mixtures and adverse pregnancy outcomes [68, 69].

#### Application of mixture methods on overall effects of POP mixtures

The most common modeling strategies of estimating overall effects of POP mixtures were dimension reduction, index modeling, response-surface modeling and latent variable models. Among these common strategies, response-surface modeling is the most used strategy to estimate the overall effects of POP mixtures. Response-surface methods allow flexible modeling between complex high-dimensional mixture components and health outcomes and can capture non-linear and non-addictive overall effects of POP mixtures [70]. BKMR, as the most used methods under the response-surface modeling group, is a semi-parametric and supervised method, which provides estimates of overall effects in risks at a particular quantile vs. the median [22]. BKMR offers several additional features in estimating overall impact of environmental pollutants mixtures. BKMR includes both the component-wise and hierarchical variable selection functions, allowing incorporation of prior knowledge of grouping in chemicals to assess single or multiple POP class chemical relationships with health outcomes [71]. BKMR also provides qualitative assessment of chemical-chemical interactions. However, response-surface modeling such as BKMR is usually computationally expensive and needs large sample sizes to ensure statistical power. It is also relatively difficult to interpret the overall effects estimates and compare these across studies.

Previous review studies on mixture methods encouraged the complementary use of multiple mixture methods [20, 21, 72]. We reported 55 studies utilizing two or more mixture methods in their analysis. The most frequently applied combination of modeling strategy is response-surface modeling with index modeling, using BKMR and WQS or quantile g-computation. Index modeling is easy to implement and interpretable. Overall effects are interpreted as effect estimates of weighted index, but they make strong assumptions in which the mixture effects are additive and exposure-response relationships are linear [32]. The main difference between WQS and quantile g-computation is WQS estimates overall effects based on prior-hypothesized directions (all positive or all negative). More recent expansions on WQS such as grouped WQS and Bayesian grouped WQS relaxed on this assumption and quantile g-computation did not have a pre-specified hypothesis on components effect directions [73, 74]. The overall effects of POP mixtures were widely hypothesized to be detrimental. Consequently, researchers need to exercise discretion in determining whether to enforce directional consistency in the modeling of the overall effects of POP mixtures.

Based on the complexity and computational demands associated with BKMR, it is recommended to prioritize index modeling for initial analyses [75]. Although BKMR offers flexible assessments of environmental pollutant mixtures and allows for component-wise and hierarchical variable selection, its implementation requires large sample sizes and significant computational resources. Additionally, interpreting the overall effects and comparing them across studies can be challenging. Conversely, index modeling is more straightforward to implement and interpret, making it a practical first step. Given these considerations, starting with index modeling before employing BKMR can streamline the analysis process while maintaining robustness in assessing environmental health impacts.

In addition, apart from the dimension reduction methods, methods as index modeling, response-surface modeling and latent variable models require prior knowledge or researcher’s threshold choice of mixture components included in modeling. The choice of chemicals in supervised methods is usually informed by the exposure assessments or findings from toxicological studies. Unsupervised methods such as shrinkage approaches like LASSO, WQS or traditional dimension reduction methods can help with the decision of choosing chemical components [21, 32]. Researchers need to make decisions on whether to use unsupervised methods or supervised methods with prior knowledge for chemical combinations they would like to estimate the overall effects of POP mixtures in the outcome of interest.

#### Reporting standard

The reporting standards for estimating the overall impact of mixtures of POPs in epidemiological studies exhibit notable heterogeneity. In our case study on PFAS mixtures and birth weight, eight studies focus on individual PFAS within the mixture. In contrast, eleven studies adopted a more comprehensive exposome-based strategy, considering multiple classes of POPs simultaneously or other chemical stressors in addition to PFAS. Moreover, nine different mixture methods were used across the studies we included in the case study, which hinders direct comparisons across studies and poses challenges for synthesizing evidence across the literature.

Studies with index models offer relatively interpretable effect estimates of weighted index of combined POP mixtures. However, the issue arises as percentiles in each study population differ, requiring conversion to absolute numbers for improved comparability. Similarly, in response-surface modeling methods like BKMR, estimates are usually presented in percentages of exposures, posing a similar challenge for cross-study comparisons. Additionally, in dimension reduction models like PCA, although estimates are easier to compare across studies, the difficulty lies in inferring realistic chemical mixtures in human bodies due to the ambiguous nature of cluster compositions. To enhance reporting practices in overall effects of POP mixtures, some practices can be implemented across studies. For example, while BKMR allows exploration of chemical-chemical interaction and non-linear overall effects, it is often observed that the dose-response relationship is described qualitatively rather quantitatively. To address this, it is recommended to report the group Posterior inclusion probabilities (PIPs) of each pre-defined groups as the overall impacts of sub-groups of POP mixtures. Additionally, reporting POP mixtures concentrations at each percentile of the analytical samples for both surface modeling and index modeling will greatly enhance the interpretability of effect size on health outcomes and will ensure that future meta-analyses can more effectively compare and synthesize findings of the overall effects POP mixtures on health outcomes.

#### Incorporation of chemical-chemical interactions

The current modeling strategies of estimating overall effects of POP mixtures emphasized on the cumulative burdens of mixtures of interest. It is also important to focus on whether the overall effects of POP mixtures have any departure from the additive effect of individual POP components as well as identification of specific interactions among POP components across the entire dose range [76–78]. In the case study of PFAS mixtures on birth weight, we observed that very few studies have identified interactions between chemical stressors. In fact, the statistical power to detect interactions is usually low, particularly with multiple chemical stressors, making the discovery of chemical-chemical interactions challenging using common modeling strategies [32, 79, 80]. BKMR can provide qualitative measures of chemical interaction effects on health outcomes. Boosted regression trees (BRT) can estimate H-statistics—unique measures of interaction relevance—and rank the importance of pairs of chemicals [81, 82]. These methods can be coupled with regression models or mixture methods with index modeling strategies to formally evaluate the interaction effects of selected interactions. Additionally, machine learning methods such as signed iterative random forest (SiRF) coupled with WQS can provide an alternative solution to quantitatively searching for synergistic effects of chemical stressors on health outcomes [76].

### Strengths and limitations

This study is the first scoping review on the overall effects of POP mixtures using mixture methods. The review offers a comprehensive perspective on the existing literature concerning the overall effects of POP mixtures and identifies challenges associated with the application of statistical methods in epidemiological studies related to POP mixtures. We included all studies with biomonitoring measurement of POP mixtures and all health outcomes to showcase the differences in applications of mixture methods. This review has certain limitations. The literature uses mixed terminology to refer to “overall effects of POP mixtures”, having different nomenclatures such as mixture effects, collective effects or cocktail effects as the overall impact of chemical mixtures. The choice of terminology might have inadvertently excluded relevant studies estimating overall effects of POP mixtures. However, our rigorous two-stage search approach was designed to comprehensively capture studies utilizing both traditional and novel mixture methods, which attenuated the risk of overlooking relevant studies. Our study also did not focus on synergistic or antagonistic effects of POP mixtures from the toxicology perspective. Future research should investigate potential interaction effects among individual POP components in relation to the overall effects of POP mixtures. In addition, while estimates of the overall effects of POP mixtures focus on population-level impacts, future mixture methods analysis should also consider individual-level repeated measured POP mixtures under the exposome framework to help developing precision environmental health interventions.

## Conclusion

This scoping review synthesized the existing body of literature on the application of statistical methodologies for estimating the overall effects of POP mixtures. Our comprehensive analysis sheds light on the current state of knowledge in this domain and highlights the methodological challenges that persist in epidemiological studies dealing with POP mixtures. By offering a holistic view, we hope to guide future research endeavors, encouraging a more nuanced understanding of the complexities involved in assessing the overall effects of POP mixtures.

## Supplementary information

Supplementary_tables_S1-S4

Supplementary_tables_S5-S6

## Data Availability

See the supplementary for all included journal studies.
