# Supplementary material for: Applications of mixture methods in epidemiological studies investigating the health impact of persistent organic pollutants exposures: a scoping review
Source: J Expo Sci Environ Epidemiol. 2024 Sep 10;35(4):522–34. doi: 10.1038/s41370-024-00717-3 (PMC11891089; doi:10.1038/s41370-024-00717-3)
Supplement: Supplementary file 1 — Supplementary_tables_S1-S4 [file 41370_2024_717_MOESM1_ESM.docx]

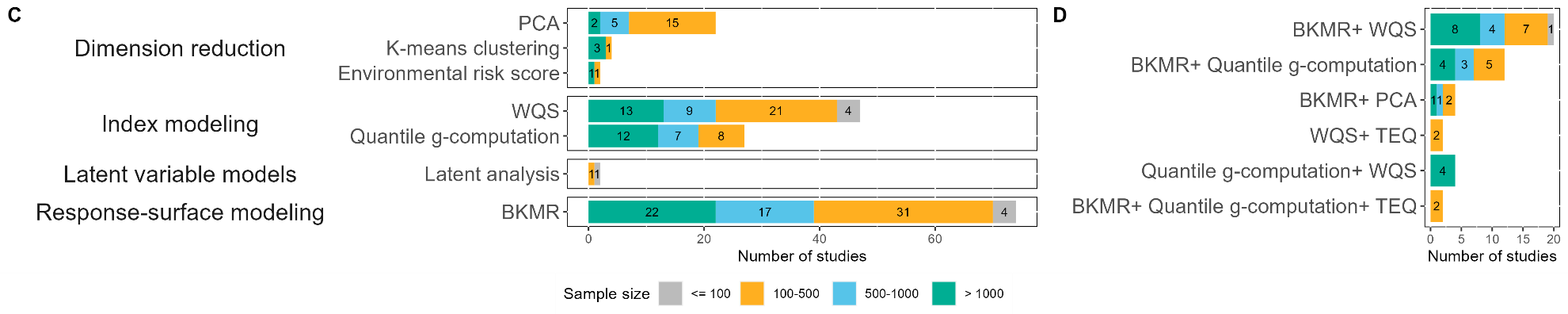


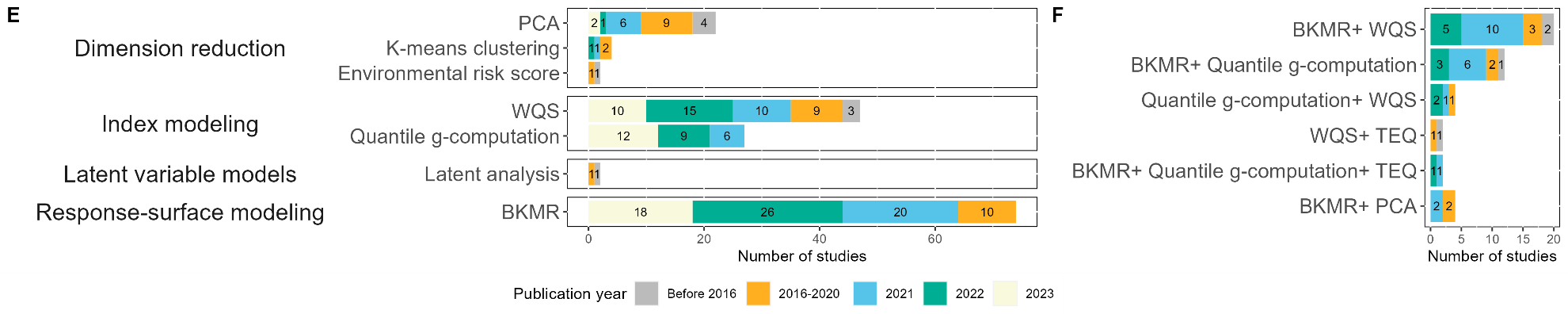


**Figure S1:** Summary of Mixture methods used in overall effect estimation from journal articles.

Panel C, D are the frequency of mixture methods in articles that applied one mixture method grouped by modeling strategy and by C) Sample size, D) publication year; Mixture methods appeared only once in articles were excluded.

Panel E, F are the frequency of mixture methods combination in articles that applied two or more mixture methods categorized by E) Sample size, F) publication year; Methods combination appeared only once in articles were excluded.

Abbreviation: PCA: Principal component analysis; WQS: Weighted quantile sum; BKMR: Bayesian kernel machine regression; TEQ: toxicity equivalent quantity

**Table S1:** Search strategies of Embase and PubMed

### PubMed search term:

("Persistent Organic Pollutants"[Mesh] OR “persistent organic pollutant*” OR Perfluoroalkyl OR Polyfluoroalkyl OR Perfluorinated OR polyfluorinated OR perfluoro* OR polyfluoro* OR PFAS* OR PFOS OR ((perfluorooctanesulfonic OR perfluorooctane sulfonic) AND acid) OR “perfluorooctane sulfonate” OR PFOA OR “perfluorooctanoic” acid OR perfluorooctanoate OR PFHxS OR ((perfluorohexane sulfonic OR perfluorohexanesulfonic) AND acid) OR “perfluorohexane sulfonate” OR perfluorohexanesulfonate OR PFNA OR “perfluorononanoic acid” OR perfluorononanoate OR GenX OR “hexafluoropropylene oxide dimer acid” OR PFOSA OR “perfluorooctane sulfonamide” OR PFUnDA OR “perfluorodecanoic acid” OR perfluoroundecanoate PFDA OR “perfluorodecanoic acid” OR perfluorodecanoate OR PFBS OR “perfluorobutane sulfonic acid” OR “perfluorobutane sulfonate” OR "perfluorooctanoic acid"[Supplementary Concept] OR "perfluorooctane sulfonic acid"[Supplementary Concept] OR OCP OR "organochlorine pesticide*" OR “hydrocarbons, fluorinated”[Mesh] OR "hydrocarbons, fluorinated" OR "fluorinated hydrocarbon*" OR "Hydrocarbons, chlorinated"[Mesh] OR "hydrocarbon, chlorinated" OR "chlorinated hydrocarbon*" OR "aldrin"[Mesh] OR "aldrin" OR "carbon tetrachloride"[Mesh] OR "carbon tetrachloride" OR "chlordan"[Mesh] OR "chlordan" OR "chlorobenzenes"[Mesh] OR "chlorobenzene*" OR "chlorofluorocarbons"[Mesh] OR "chlorofluorocarbon*" OR "dichloroethylenes"[Mesh] OR "dichloroethylene*" OR "dieldrin"[Mesh] OR "dieldrin" OR "endrin"[Mesh] OR "endrin" OR "heptachlor"[Mesh] OR "heptachlor" OR "methoxychlor"[mesh] OR "methoxychlor" OR "mirex"[Mesh] OR "mirex" OR "ddt"[Mesh] OR ddt OR dde OR "Dichlorodiphenyl Dichloroethylene"[Mesh]OR "endosulfan"[Mesh] OR "endosulfan" OR "toxaphene"[Mesh] OR "toxaphene" OR “camphechlor" OR hcb OR "hexachlorobenzene"[Mesh] OR hch OR "hexachlorocyclohexane"[Mesh] OR "halogenated Diphenyl Ethers"[Mesh] OR "halogenated diphenyl ether*" OR "polychlorinated biphenyls"[Mesh] OR “polychlorinated biphenyl*” OR pcb OR "polychlorinated dibenzodioxins"[Mesh] OR pcdd OR "Dibenzofurans, Polychlorinated"[Mesh] OR pcdf OR "polybrominated diphenyl ether*" OR pbde OR "Flame Retardants"[Mesh] OR "flame retardant*")
AND
(“mixture*” OR "chemical mixture*" OR "overall effect*" OR "overall association*" OR "overall exposure*" OR "cumulative effect*" OR "combined effect*" OR "joint impact*" OR “joint exposure*” OR “joint effect*” OR "multi-pollutant" OR multipollutant OR WQS OR "weighted quantile" OR bkmr OR "Bayes Theorem"[mesh] OR “Bayesian” OR "g-computation" OR "g computation" OR pca OR “principal component*” OR “clustering” OR “Exposure continuum mapping” OR “latent class” OR “latent profile” OR FIN OR "factor analysis" OR BSSVI OR "SGP-MPI" OR "RH-WQS" OR "Mult DLAG" OR MatchAlign OR LWQS OR "riPS OR DLMtree"OR DAG OR "directed acyclic graph*" OR BVSM OR BMIM OR "bayesian multiple index model" OR BKMR-DLM OR "BKMR-CMA" OR "Bayes Tree Pairs" OR ACR OR "Acceptable Concentration Range model" OR SPAMTREE OR "Spatial Mutivariate Trees" OR FOTP OR TEV OR SCC OR "Spatiotemporal case-crossover" OR "GL-GPs" OR BDS OR "Bayesian Data Synthesis")
NOT (animals [mesh] NOT (humans [mesh] AND animals[mesh]))

Filters: 2011-2023

**Embase search terms:**

‘persistent organic pollutant’/exp OR ‘persistent organic pollutant*’ OR Perfluoroalkyl OR Polyfluoroalkyl OR perfluorinated OR polyfluorinated OR perfluoro OR polyfluoro* OR PFAS* OR PFOS OR ((perfluorooctanesulfonic OR perfluorooctane sulfonic) AND acid) OR ‘perfluorooctane sulfonate’ OR PFOA OR ‘perfluorooctanoic acid’ OR ‘perfluorooctanoate’ OR PFHxS OR ((perfluorohexane sulfonic OR perfluorohexanesulfonic) AND acid) OR ‘perfluorohexane sulfonate’ OR perfluorohexanesulfonate OR PFNA OR ‘perfluorononanoic acid’ OR perfluorononanoate OR GenX OR ‘hexafluoropropylene oxide dimer acid’ OR PFOSA OR ‘perfluorooctane sulfonamide’ OR PFUnDA OR ‘perfluorodecanoic acid’ OR ‘perfluoroundecanoate PFDA’ OR ‘perfluorodecanoic acid’ OR perfluorodecanoate OR PFBS OR ‘perfluorobutane sulfonic acid’ OR ‘perfluorobutane sulfonate’ OR ocp OR ‘chlorinated hydrocarbon’/exp OR ‘chlorinated hydrocarbon*’ OR ‘organochlorine pesticide*’ OR ‘organochlorine pesticide’/exp OR ‘organochlorine insecticide’/exp OR ‘organochlorine insecticide*’ OR ‘organochlorine’ OR ‘fluorinated hydrocarbons’/exp OR ‘fluorinated hydrocarbon*’ OR ‘aldrin’/exp OR aldrin OR ‘carbon tetrachloride’/exp OR ‘carbon tetrachloride’ OR ‘chlordan’/exp OR chlordan OR ‘chlorobenzene’/exp OR chlorobenzene* OR ‘chlorofluorocarbon’/exp OR chlorofluorocarbon* OR ‘vinylidene chloride’/exp OR ‘dichloroethylene*’ OR ‘dieldrin’/exp OR dieldrin OR ‘endrin’/exp OR endrin OR ‘heptachlor’/exp OR heptachlor OR ‘methoxychlor’ OR ‘methoxychlor’/exp OR ‘mirex’/exp OR ‘mirex’ OR ‘ddt’/exp OR DDT OR ‘dde’/exp OR DDE OR ‘endosulfan’ OR ‘endosulfan’/exp OR campheclor/exp OR campheclor OR toxaphene OR ‘hexachlorobenzene’/exp OR hexachlorobenzene OR HCB OR HCH OR ‘hexachlorocyclohexane’/exp OR ‘diphenyl ether derivative’/exp OR ‘halogenated diphenyl ether*’ OR ‘polychlorinated biphenyls’/exp OR ‘polychlorinated biphenyl*’ OR pcb OR ‘polychlorinated dibenzodioxin’/exp OR pcdf OR pcdd OR ‘polychlorinated dibenzofuran’/exp OR pbde OR ‘polybrominated diphenyl ethers’ OR ‘flame retardant’/exp OR ‘flame retardant*’
AND

mixture* OR ‘chemical mixture*’ OR ‘overall effect*’ OR ‘overall association*’ OR ‘overall exposure*’ OR ‘cumulative effect*’ OR ‘combined effect*’ OR ‘joint impact*’ OR ‘joint exposure*’ OR ‘joint effect*’ OR ‘multi-pollutant’ OR multipollutant OR wqs OR ‘weighted quantile’ OR bkmr OR Bayesian OR ‘Bayes theorem’/exp OR ‘g-computation’ OR ‘g computation’ OR pca OR ‘principal component*’ OR clustering OR ‘exposure continuum mapping’ OR ‘latent class’ OR ‘latent profile’ OR FIN OR ‘factor analysis’ OR BSSVI OR SGP-MPI OR ‘RH-WQS’ OR ‘Mult DLAG’ OR MatchAlign OR LWQS OR GriPS OR DLMtree OR DAG OR ‘directed acyclic graph’ OR BVSM or BMIM OR ‘Bayesian Multiple index model’ OR ‘BKMR-DLM’ OR ‘BKMR-CMA’ OR ‘Bayes Tree Pairs’ OR ACR OR ‘Acceptable Concentration Range model’ OR SPAMTREE OR ‘Spatial Mutivariate Trees’ OR FOTP OR TEV OR SCC OR ‘Spatiotemporal case-crossover’ OR ‘GL-GPs’ OR BDS OR ‘Bayesian Data Synthesis’
[embase]/lim NOT ([embase]/lim AND [medline]/lim) AND

('article'/it OR 'erratum'/it OR 'preprint'/it OR 'review'/it)

NOT ([animals]/lim NOT [humans]/lim)

AND [2011-2023]/py

**Table S2:** Inclusion and exclusion criteria

|  | Inclusion | Exclusion |
| --- | --- | --- |
| Population (P) | Human subjects |  |
| Exposure (E) | - Evaluated at least three individual POPs based on NIEHS definition of mixture^26^. - Direct measurements via biomonitoring | - Measured through air, food, drinking water, occupational settings, or direct administration |
| Outcome (O) | Any health outcomes |  |
| Results (R) | Used mixture methods selected *a priori*:   - WQS - BKMR - quantile g-computation - PCA - factor analysis - clustering analysis - exposure continuum mapping - latent class/profile analysis - other Bayesian approaches related to environmental mixture overall effects - Novel methods from the PRIME workshop ^15^ | - used only summation measures of POPs - Used methods selected a prior but did not provide overall effects of POPs. - Only used methods that are based on toxicology results simulated from animal models or cell lines. Eg. Toxic equivalency factors and TEQ, androgen receptor TEQ |
| Additional criteria | - Observational epidemiologic studies - No language restriction - Journal articles published after 2011 | - In silico studies - Gray literature - Reviews - Pre-prints - Conference proceedings - Editorials - Websites - Chapters in textbooks |

Abbreviation: NIEHS: The national institute of environmental health sciences; PCA: Principal component analysis; WQS: Weighted quantile sum; BKMR: Bayesian kernel machine regression; TEQ: toxicity equivalent quantity

**Table S3:** Data classifiers for the data extraction

|  | Definition |
| --- | --- |
| Title/author with publication date |  |
| Health outcome categories | Selected from: Body weight, Size & Growth, Cancer, Cardiometabolic, Cardiovascular, Dermal, Endocrine, Hepatic, Immune, Metabolic, Mortality, Musculoskeletal, Nervous, Reproductive, Respiratory, Systemic biomarkers, and Urinary.  Note: Biomarkers were categorized based on their association with specific health outcomes; for example, liver biomarkers were classified under "Hepatic." Conversely, biomarkers indicative of nonspecific processes, which are linked to multiple health outcomes, were designated as "systemic biomarkers."  For simplicity and statistical clarity, some health outcomes that span multiple categories have been consolidated into a single category. Details of this classification are provided in Table S4. |
| Study country | The country where the participants were recruited |
| Study site | Study city or state/province where the participants were recruited if provided |
| Study design | Cohort/case-control/cross-sectional |
| Study population | Description of the study population, including the recruitment year, cohort name if applicable; for case-control studies, cases and control demographic statistics and recruitment strategy |
| Sample size | Analytic sample for mixtures if provided, if not, will report the sample size used in general population; cases and control sample sizes were reported separately in case-control studies |
| Age | Mean of age for the population of interest (medians were reported if means were not found), ages of cases and controls were documented separately for case-control studies. |
| Exposure matrix | Biological samples used for exposure assessment |
| POP exposures included in each mixture methods | Individual chemicals used in each mixture methods or models if multiple mixture methods or models were reported in journal articles |
| Chemical list | Full list of chemical compounds names which harmonized between journal articles according to our chemical list (see list in Table S5) |
| Outcome(s) studied | Main outcomes reported as relation to POP mixtures |
| Mixture methods | Mixture methods used to estimate overall effects of POP mixtures |
| Covariates | Covariates used for estimating overall effects of POP mixtures. If not identified from mixture models, we extracted covariates used for single-pollutant models reported in the same journal articles |

**Table S4:** Health outcome category classification based on included journal articles.

| Health outcome category classifier | Health Outcomes |
| --- | --- |
| Body weight, Size and Growth | Anogenital distance, birth-related measures (birth weight, birth length, ponderal index, growth hormone, gestational age, gestational weight gain, gestational weight gain rate, head circumference), BMI, waist circumstance |
| Cancer | Thyroid cancer, breast cancer, breast cancer metastasis and tumor size |
| Cardiometabolic | Two or three combinations of total cholesterol, high-density lipoprotein cholesterol, |
| Cardiovascular | Hypertensive disorder of pregnancy, blood pressure levels, cardiovascular disease, heart rate variability parameters, carotid intima-media thickness, acute coronary syndrome, cardiac intraventricular and posterior wall thickness, relative wall thickness |
| Dermal | Chronic spontaneous urticaria |
| Endocrine | Thyroid hormone levels, hypothyroidism and any thyroid disease, nodular goiter, sex steroids |
| Hepatic | Liver injury, liver function biomarkers: |
| Immune | Asthma and eczema symptoms, change in inflammatory biomarkers (cytokines), immunoglobulin, common cold, antibodies of diseases, staphylococcus aureus colonization |
| Metabolic | Metabolic syndrome, gestational diabetes mellitus (GDM),type-2 diabetes, dyslipidemia, hyperglycemia, hypertriglyceridemia, glucose homeostasis, lipids(high-density lipoprotein (HDL-C), low-density lipoprotein (LDL-C), total cholesterol (TC), total triglycerides (TG)), adipokines, glucose tolerance, fasting glucose, fasting insulin, HOMA-IR, Matsuda index |
| Mortality | Mortality |
| Musculoskeletal | Areal bone mineral density (aBMD), bone mineral content (BMC) |
| Nervous | ADHD, Autism spectrum disorder (ASD), depression, anxiety, neurobehavioral scores, IQ, brain-derived neurotrophic factor, inhibition, internalizing symptoms, sleep quality, reading skills, neural tube defects, smoking and drinking behaviors, survival of ALS, walking speed, working memory |
| Respiratory | Cardiorespiratory fitness from maximal oxygen consumption, fractional exhaled nitric oxide level, chronic obstructive pulmonary disease prevalence |
| Reproductive | Testosterone, miscarriage, incident natural menopause, endometriosis, reproductive parameters, preterm birth, endometriosis, anti-mullerian hormone, basal antral follicle count, ovarian sensitivity index, semen quality, semen volume, infertility, gestational age at delivery, time to pregnancy, early menarche, IVF-EV indicators, postpartum hemorrhage, gestational anemia, diminished ovarian reserve |
| Systemic biomarkers | Oxidative stress biomarkers, DNA methylation, vitamin D biomarkers, telomere length, retinol and RBP4 levels, micro-RNAs, hemoglobin and hematocrit during pregnancy, allostatic load |
| Urinary | Hyperuricemia, chronic kidney disease, albuminuria, uric acid levels, glomerular filtration rate (eGFR) |
